# Observing flaming or trolling online: Prevalence in russian youth and adolescents and relationship to tolerance and aggression

**DOI:** 10.1192/j.eurpsy.2021.553

**Published:** 2021-08-13

**Authors:** G. Soldatova, S. Chigarkova, A. Koshevaya, E. Rasskazova

**Affiliations:** Faculty Of Psychology, Lomonosov Moscow State University, Moscow, Russian Federation

**Keywords:** adolescents, cyberaggression, flaming, trolling

## Abstract

**Introduction:**

Cyberaggression including the most wide-spread variants of flaming (O’Sullivan, Flanagin, 2003; Voggeser et al., 2017) and trolling (Buckels et al., 2018) is affecting mental health of adolescents and youth although it could be (Kowalski, 2014; Wright, Wachs, 2020).

**Objectives:**

The aim was to study prevalence of flaming and trolling experience in Russian youth and adolescents and its relationship to general aggression and tolerance.

**Methods:**

525 adolescents 12-13 years old, 1029 adolescents 14-17 years old, 736 youth 18-30 years from 8 Federal regions in Russia appraised their experience of flaming or trolling online (as initiators, victims and observers) using vignettes. 1105 parents of adolescents appraised whether their children experienced flaming or trolling online. Then they filled Aggression Questionnaire (Buss, Perry, 1992) and Tolerance Index (Psychodiagnostics…, 2008).

**Results:**

More than one-half of adolescents (51-58% in 12-13 years old and 64% in 14-17 years old) and youth (45-69%) reported experience of flaming and trolling online, mostly as observers (32-65%). Parents accurately appraised flaming experience in their children but underestimated trolling experience (p<.05). Adolescents and youth observing flaming online report higher hostility, anger and physical aggression (F=17.8-28.3, p<.01, η²=.02) while lower social tolerance (F=4.27, p<.05, η²=.01). In adolescents observing trolling online these effects are stronger than in youth observing trolling online (interaction: F=5.68, p<.05, η²=.01).

**Conclusions:**

Observing trolling and flaming online is related to higher aggression and low tolerance in adolescents and youth and for adolescents the relationship is stronger. The reported study was funded by RFBR, project 20-013-00857.

**Conflict of interest:**

The reported study was funded by RFBR, project 20-013-00857.

